# Patient and tumor factors contributing to distant metastasis in well-differentiated thyroid cancer: a retrospective cohort study

**DOI:** 10.1186/s40463-020-00469-8

**Published:** 2020-11-16

**Authors:** Usman Khan, Ayham Al Afif, Abdullah Aldaihani, Colin MacKay, Matthew H. Rigby, Murali Rajaraman, Syed Ali Imran, Martin J. Bullock, S. Mark Taylor, Jonathan R. B. Trites, Robert D. Hart

**Affiliations:** 1 Faculty of Medicine, Dalhousie University, 1459 Oxford Street, Halifax, Nova Scotia B3H 4R2 Canada; 2 Division of Otolaryngology – Head and Neck Surgery, Dalhousie University, Dickson Building, QEII Health Sciences Centre, 5820 University Avenue, Halifax, Nova Scotia B3H 2Y9 Canada; 3 Department of Radiation Oncology, Dalhousie University, Nova Scotia Cancer Centre, 5820 University Avenue, Halifax, NS B3H 1V7 Canada; 4 Division of Endocrinology & Metabolism, Department of Medicine, Dalhousie University, 1276 South Park Street, Suite 7-North-048 Victoria Building, Halifax, NS B3H 2Y9 Canada; 5 Department of Pathology, Dalhousie University, DJ Mackenzie Building, 5788 University Avenue, Halifax, Nova Scotia B3H 2Y9 Canada; 6 Division of Otolaryngology – Head and Neck Surgery, University of Calgary, ENT Clinic, 1820 Richmond Road SW, Calgary, AB T2T 5C7 Canada

**Keywords:** Distant metastasis, Thyroid cancer, Well differentiated thyroid cancer, Metastatic thyroid cancer, Thyroid cancer survival

## Abstract

**Background:**

Distant metastasis in thyroid cancer significantly reduces survival in patients with well-differentiated thyroid carcinoma (WDTC). There is limited information available to clinicians regarding pathological features that confer a higher risk of distant metastasis (DM). This study aimed to identify patient and tumor factors that were associated with the development of DM over time in patients with WDTC.

**Methods:**

A retrospective cohort analysis of patients with WDTC (*n = 584*) at our institution was performed between 2007 and 2017. A total of 39 patients with DM and 529 patients with no DM (NDM) were included. Patient demographics, tumor characteristics and patient survival were compared between the DM and NDM groups using a univariate analysis. Multivariate Cox-proportional hazards model was used to evaluate the risk of developing distant metastasis over time. Kaplan-Meier analysis was used to compare survival between the DM and NDM groups.

**Results:**

Distant metastasis had a substantial impact on disease-specific survival (DSS) at 5 and 10-years in the DM group; 71.0% (SE 8.4%) and 46.9% (SE 11.6%) respectively, compared to 100% survival in the NDM group (*p* < 0.001). The DM group had significantly higher proportions of males, lymphovascular invasion (LVI), nodal metastasis (NM), large tumor size (TS), extrathyroidal extension (ETE), positive resection margins, multifocality, follicular thyroid cancer (FTC), tall cell variant of papillary thyroid cancer (PTC), and Hurthle cell carcinoma (HCC), when compared to the NDM group (*p < 0.05*). A TS ≥ 2 cm (Hazard Ratio (HR) 1.370), NM (HR 3.806) and FTC (HR 7.068) were associated with a significantly increased hazard of developing distant metastasis in patients with WDTC.

**Conclusions:**

TS ≥ 2 cm, NM and FTC are associated with a significantly increased propensity for developing DM in our cohort of WDTC patients.

**Graphical abstract:**

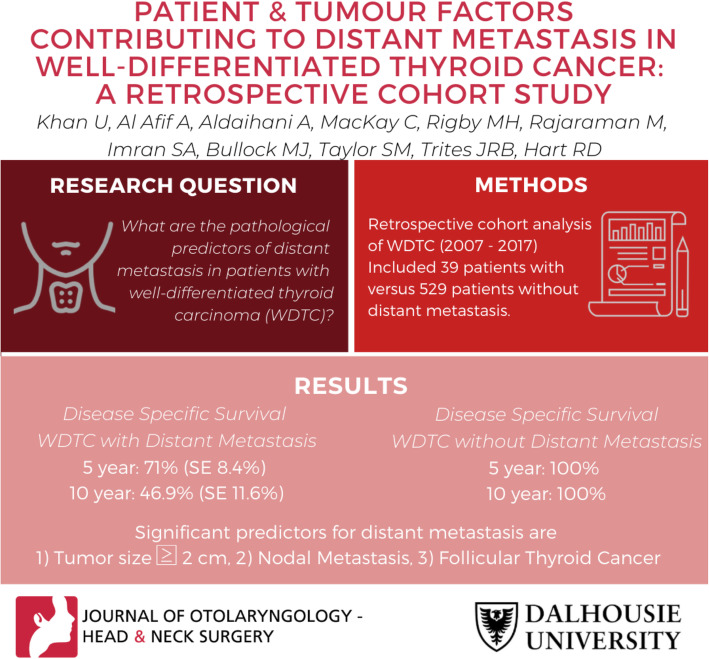

## Background

The incidence of well-differentiated thyroid cancer (WDTC) has increased substantially in recent years, largely owing to a more widespread use of sensitive diagnostic techniques [1]. Improved multidisciplinary treatment regimens have enhanced patient survival rates, estimated at 95% and higher over 10 years [2]. Although distant metastasis (DM) in WDTC remains uncommon, it is associated with a significantly lower disease-specific survival (DSS), estimated at 30–50% (5–10 years), compared to patients with no DM [3–5].

While several studies have reported the prognosticators of survival [5–10], local recurrence and mortality [6, 11, 12] in WDTC, there remains a lack of information regarding the pathological predictors of DM in this population. Specifically, there is limited information regarding the contributions of aggressive pathological subtypes of WDTC [5, 6] in the development of DM, such as non-classical subtypes of papillary thyroid carcinoma (PTC) (tall cell, oncocytic variants), follicular thyroid carcinoma (FTC) and Hurthle cell carcinoma (HCC). The impact of pathological features such as tumor size, lymphovascular invasion, nodal metastasis, multifocality and positive resection margins also remain controversial [13–16]. While some studies have investigated DM in WDTC, there are limited studies utilizing a statistical model that evaluates the development of DM over time for clinically-relevant pathological features.

In this study, we investigated our cohort of WDTC patients to identify patient and pathological factors that are associated with the development of DM over time. Given that PTC is the most common thyroid malignancy with the most favorable clinical outcomes, a separate subgroup analysis of patients with PTC was also conducted. We also report on one of the largest samples of patients with WDTC and DM in the Canadian literature. The overall aim of this study was to better understand the pathological predictors for developing DM in thyroid carcinoma, which will ultimately better identify, and inform the management of, patients with WDTC.

## Methods

The interdisciplinary thyroid cancer database (ITCD) is a computerized registry at our institution which prospectively collects information on all patients referred to the thyroid oncology clinic. A thorough search of the ITCD at our institute from 2007 to 2017 identified 584 patients with a diagnosis of thyroid carcinoma, confirmed on post-operative pathology. Patients with medullary thyroid carcinoma and anaplastic thyroid carcinoma were excluded (*n* = 16). A total of 39 patients with a diagnosis of thyroid carcinoma and distant metastasis (DM) were identified. Patients were included whether DM was diagnosed on initial presentation or throughout the course of treatment. The remaining 529 patients were included as the non-distant metastasis group (NDM). A diagnosis of DM was confirmed with a combination of biopsy of the site of metastasis, positron emission tomography (PET) scan and/or computed tomography (CT), and Iodine-131 scan.

Variables analyzed included age at diagnosis, sex, pathological type of thyroid cancer, histological subtype, primary tumor size (cm), focality, lymphovascular invasion (LVI), margin status, extrathyroidal extension (ETE) and nodal metastasis (NM). Multifocal disease was defined by the presence of ≥3 tumor foci. This was based on findings by Al Afif et al. where ≥3 foci led to a significantly increased propensity for NM [17]. We also compiled data on therapeutic strategies of patients including surgical resection of distant metastasis, the number of radioactive iodine (RAI) treatments, and whether or not external beam radiation therapy (EBRT) was delivered. Cumulative and disease-specific survival were computed for both patient groups, followed by an analysis of variables contributing to the risk of developing DM. A tumor size of 2 cm was associated with increased mortality in previous articles and was therefore used as a cut-off for our hazard model analysis of DM [16, 18, 19].

### Statistical analysis

Univariate analysis was performed using Fisher’s exact test or Chi Square test for categorical variables, and Mann-Whitney U test for continuous variables. Cumulative and disease-specific survival plots were generated using the Kaplan-Meier method. Differences in survival were tested using log-rank comparisons. The multivariate Cox proportional hazard model was utilized to evaluate the association of variables with the development of DM over time. All statistical analysis was conducted on SPSS (IBM SPSS Statistics 25). A *p-value* of < 0.05 was considered significant for all tests.

## Results

### Demographics and tumor pathology

Clinical and pathological variables were analyzed in both the NDM (*n* = 529) and DM (*n* = 39) patient groups (Table 1). Patients with DM, when compared with those in the NDM group, were older at diagnosis (55 years ±16.6 vs. 50 years ±14.4 *p* = 0.026); were more likely to be male (46% vs. 24%, *p* = 0.002); had larger mean tumor size (4.5 cm ± 3 vs 2.0 cm ± 1.7, *p* < 0.001); had multifocal tumors (69.2 vs. 30.1; *p* < 0.001); had LVI (81% vs 29%; *p* < 0.001); had NM (62.9% vs. 26.1%; *p* < 0.001); had ETE (48.4% vs 23.4%; *p* = 0.002) and positive tumor resection margins (46.9% vs. 26.5%; *p* = 0.013).

**Table 1:** Clinical and pathological characteristics of DM (*n* = 39) and NDM (*n* = 529) groups

Variable	DM	NDM	***p-value***
Sex (% M:F)	46:54	24:76	0.002
Average age (SD) (years)	55 (16.6)	50 (14.4)	0.026
Average tumor size (SD) (cm)	4.5 (3.0)	2.0 (1.7)	< 0.001
Multifocal (%)	69.2	30.1	< 0.001
Positive margins (%)	46.9	26.5	0.013
LVI (%)	81.2	29.4	< 0.001
Nodal metastasis (%)	62.9	26.1	< 0.001
ETE (%)	48.4	23.4	0.002

*DM* Distant metastasis, *NDM* Non-distant metastasis, *LVI* Lymphovascular invasion, *ETE* Extrathyroidal extension

The distribution of tumor histopathology is summarized in Table 2. PTC was the most prevalent entity in both groups with the classical subtype being commonest (46.4% in DM and 45.3% in NDM respectively; *p <* 0.05). There were fewer cases of the follicular subtype of PTC in the DM compared with the NDM group (14.3% vs 30.5%, respectively; *p =* 0.05). However, DM group compared with NDM group had a higher preponderance of tall cell variant of PTC (14.3% vs 2.5%; *p* = 0.028), FTC (12.8% vs 2.1%; *p* = 0.003) and HCC (15.4% vs 1.1% in NDM; *p < 0.001*) respectively. There was no significant difference (*p >* 0.05) in the distribution of the oncocytic subtype of PTC (7.1 and 1.8%, respectively) and micropapillary carcinoma (3.6 and 5.5% respectively) between the DM and NDM groups.

**Table 2:** Distribution of tumor histopathology in DM and NDM patient groups

Tumor Type	DM %	NDM (%)
Papillary Thyroid Carcinoma*	71.8	96.7
*Classical subtype*	*46.4*	*45.3*
Tall Cell subtype*	*14.3*	*2.5*
*Follicular subtype*	*14.3*	*30.5*
*Oncocytic subtype*	*7.1*	*1.8*
*Microcarcinoma*	*3.6*	*5.5*
*Other or unknown*	*14.3*	*14.4*
Hurthle Cell Carcinoma*	15.4	1.1
Follicular Cell Carcinoma*	12.8	2.1

*DM* Distant metastasis, *NDM* Non-distant metastasis

* *p < 0.05*

### Clinical presentation and therapeutic strategies

Of the 39 patients with DM, 12 had evidence of DM at the time of initial presentation whereas 27 developed DM during the course of their disease (Table 3). The commonest clinical manifestations in patients with DM on initial presentation were pathological fractures in 7/12 (58%) patients and pulmonary symptoms or incidental findings of pulmonary nodules on imaging in 5/12 (42%) patients. The first site of DM was seen in the lungs in 24/39 (62%) patients; bone in 12/39 (31%); brain in 1/39 (3%); mediastinal lymph nodes in 1/39 (3%). One patient (3%) had metastasis to multiple sites simultaneously. The anatomical sites of DM based on whether it occurred on presentation or throughout the course of the disease are further delineated in Table 3. The management of patients with DM is highlighted in Table 3. All patients with DM received at least one cycle of RAI, with 17/39 (44%) receiving more than 1 cycle. EBRT was administered in 22/39 cases (56%). In patients with DM, 13/39 (33%) were offered distal metastatectomy.

**Table 3:** Clinical features and therapeutic management of patients in the DM group (*n = 39*) based on initial presentation with DM (*n = 12*) or development of DM after initial diagnosis (*n = 27*)

Variable	Presented with DM ***n*** (%)	Developed DM after diagnosis ***n*** (%)	Total ***n*** (%)
Presenting Symptom			
Pathological fracture	7 (58)	_	_
Pulmonary symptoms or incidental lung nodules	5 (42)	_	_
Presenting site of DM			
*Lung*	5 (42)	19 (70)	24 (62)
*Bone*	7 (58)	5 (19)	12 (31)
*Brain*	0 (0)	1 (3)	1 (3)
*Mediastinum*	0 (0)	1 (3)	1 (3)
*Multiple sites*	1 (3)	0 (0)	1 (3)
Multiple sites of DM	8 (67)	10 (37)	18 (46)
Treatment			
*1 RAI cycle*	12 (100)	27 (100)	39 (100)
*> 1 RAI cycle*	6 (50)	11 (41)	17 (44)
*EBRT*	10 (83)	12 (44)	22 (56)
*Metastectomy*	7 (58)	6 (22)	13 (33)

*DM* Distant metastasis, *RAI* Radioactive iodine, *EBRT* External beam radiation therapy

Univariate analysis was used to compare patients with DM on presentation with those who developed DM during the course of their treatment (Table 4). Positive margins (*p* = 0.019) and ETE (*p* = 0.032) were significantly more prevalent in the group that developed DM after diagnosis. Sex, age, tumor size, LVI, NM, tumor histology and focality were not significantly different between both groups.

**Table 4:** Univariate analysis of patients in the DM group comparing those with DM on initial presentation (*n = 12*) and those who presented after their diagnosis of thyroid cancer (*n = 27*)

Variable	***p-value***
Sex	0.999
Focality	0.061
Positive margins*	0.019
LVI	0.148
Nodal metastasis	0.258
Age at diagnosis	0.563
Size	0.253
ETE*	0.032

*LVI* Lymphovascular invasion

*p < 0.05

### Multivariate analysis

Multivariate Cox proportional hazards model was used to determine which covariates were independently associated with the development of DM over time (Table 5). Of the ten covariates tested, only tumor size ≥2 cm (Hazard Ratio (HR) = 1.37, 95% confidence interval (CI) = 1.135–1.655, *p* = 0.001), NM (HR = 3.806, 95% CI = 1.285–11.270, *p* = 0.016) and FTC (HR = 7.068, 95% CI = 1.869–26.731, *p* = 0.004) had a statistically significant increased HR for DM.

**Table 5:** Hazard ratios by Cox proportional hazards model for predicting the risk of distant metastasis in WDTC in DM and NDM patients (*n = 568*)

Variable	HR	Lower CI	Upper CI	***p***-value
Age	1.023	0.993	1.055	0.131
Sex	1.503	0.607	3.722	0.378
Follicular	7.068	1.869	26.731	0.004^†^
Hurthle	1.945	0.416	9.086	0.398
Size	1.370	1.135	1.655	0.001^†^
Focality	1.850	0.764	4.482	0.173
Positive Margins	0.974	0.374	2.540	0.957
LVI	2.488	0.719	8.613	0.15
Nodal Metastasis	3.806	1.285	11.270	0.016^*^
ETE	0.883	0.311	2.502	0.815

*LVI* Lymphovascular invasion, *ETE* Extrathyroidal extension, *CI* Confidence interval

*p < 0.05

### Subgroup analysis of papillary thyroid Cancer patients

Multivariate Cox proportional hazards model was performed to determine which covariates were independently associated with the development of distant metastasis over time in patients with PTC only (Table 6). Tumor size ≥2 cm (HR = 1.354, 95% CI = 1.113–1.649, *p* = 0.002), increasing age (HR = 1.035, 95% CI = 1.000–1.071, *p* = 0.048), multifocality (HR = 3.032, 95% CI = 1.120–8.206, *p* = 0.029) and NM (HR = 4.761, 95% CI = 1.457–15.553, *p* = 0.01) had a statistically significant increased HR for DM.

**Table 6:** Hazard ratios by Cox proportional hazards model for all DM and NDM patients with PTC (*n = 540*) to predict the risk of distant metastasis

Variable	HR	Lower CI	Upper CI	***p***-value
Age	1.035	1.000	1.071	0.048^*^
Sex	1.382	0.518	3.684	0.518
Size	1.354	1.113	1.649	0.002^*^
Focality	3.032	1.120	8.206	0.029^*^
Positive Margins	1.068	0.373	3.056	0.903
LVI	1.748	0.465	6.575	0.409
Nodal Metastasis	4.761	1.457	15.553	0.01^*^
ETE	0.942	0.278	3.189	0.923

*LVI* Lymphovascular invasion, *ETE* Extrathyroidal extension

*p < 0.05

### Survival

**Fig. 1:**
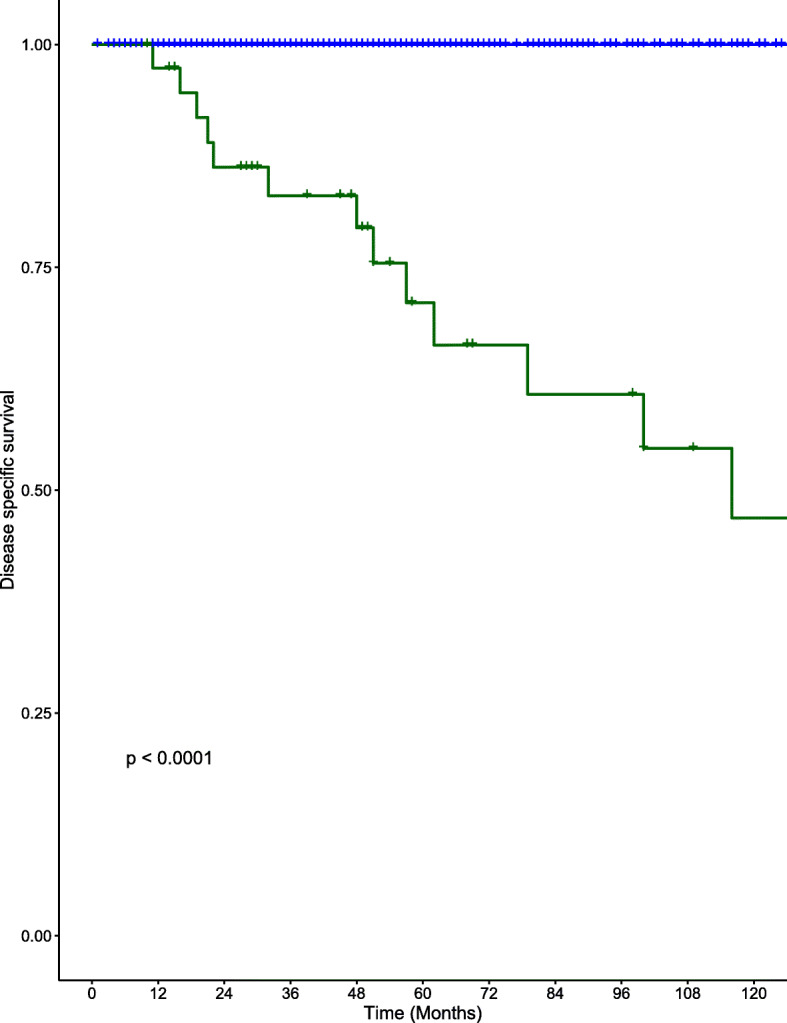
Kaplan-Meier curves for DSS in the DM (red) and NDM groups (blue) for patients with well-differentiated thyroid cancer

**Fig. 2:**
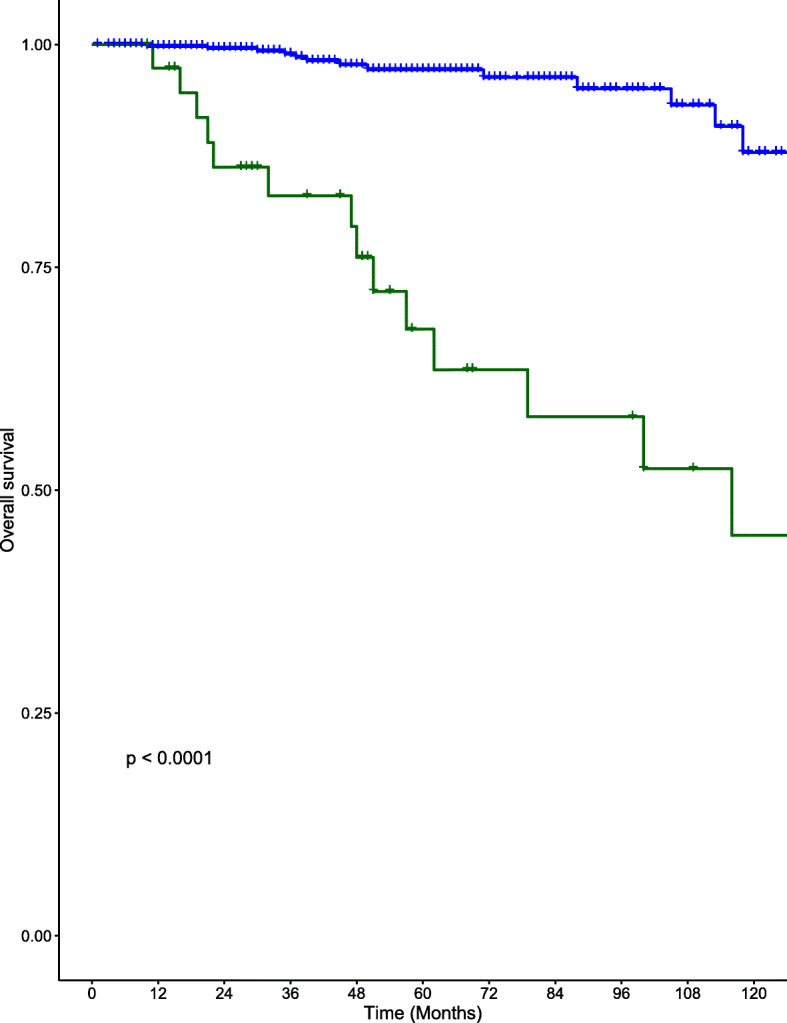
Kaplan-Meier curves for OS in the DM (red) and NDM groups (blue) for patients with well-differentiated thyroid cancer

DSS and overall survival (OS) at 10 years are shown in Figs. 1 and 2 respectively. DSS at 5- and 10-years for the DM group were 71% (standard error (SE) 8.4%) and 46.9% (SE 11.6%). DSS for the NDM group at 5- and 10 years were 100 and 100% respectively. OS at 5- and 10-years for the DM group were 68.1% (SE 8.6%) and 44.9% (11.3%). OS for the NDM group at 5- and 10 years were 97.2% (SE 1%) and 87.9% (SE 4.4%) respectively.

## Discussion

Overall, the body of evidence available to clinicians for assessing a patient’s risk for developing DM is limited. A recent meta-analysis of articles using univariate comparisons has identified male sex, advanced age, tumor size, multifocality, LVI, ETE and lymph node metastasis as risk factors for DM in WDTC [20]. Our study demonstrates similar outcomes [20]. Our data shows a significantly higher proportion of male patients, larger tumors, LVI, positive resection margins, multifocality (≥3 foci), ETE and nodal metastasis in the DM group when compared with the NDM group [5, 20]. Additionally, PTC was less prevalent in DM patients, with significantly larger proportions of FTC, when compared with the NDM group. We also investigated the impact of PTC subtype and HCC on the development of DM. We found significantly higher proportions of tall cell variant of PTC and HCC in DM patients when compared to the NDM group. A pathological diagnosis of FTC was also a significant predictor of distant metastasis in our Cox proportional hazard model analysis. Moreover, many patients with tall cell variant of PTC or HCC experienced an aggressive progression of their disease with multiple sites of DM. Our findings suggest that the disproportionally higher rates of tall cell variant of PTC, HCC and FTC in DM patients are clinically important risk factors for the development of DM.

Previous reports have mostly focused on survival prognosticators in patients who have already developed DM or performed univariate comparisons between NDM and DM patients [20]. A statistical model assessing the risk or hazard over time for developing DM for selected pathological variables in a cohort of WDTC patients is less common in the literature. In our study, the pathological variables identified in the univariate analysis were used to better assess their impact on the development of DM. Using this model, a tumor size ≥2 cm (HR = 1.4), nodal metastasis (HR = 3.8) and follicular carcinoma (HR = 7) were the only significant variables associated with an increased chance for developing DM over time. Interestingly, a tumor size ≥2 cm has also been correlated with increased mortality and adverse outcomes in patients with WDTC [16]. The age of patients, positive resection margins, ETE and sex were not associated with a risk of developing DM. Although age has been associated with improved survival in DM patients [10], our analysis did not demonstrate age as a significant factor for developing DM. This can partly be a result of wide age ranges in our dataset. However, as our model investigates the development of DM overtime, younger age may only be a prognosticator of survival once a diagnosis of DM is reached as other studies have suggested [10]. Age was a significant factor in the subgroup analysis of PTC patients alone. The literature remains divided on the predictive value of multifocality in regard to tumor recurrence, metastasis and mortality [12, 21]. The presence of ≥3 foci were not a predictor of DM in our full patient cohort despite showing a propensity for nodal metastasis in previous research [17]. However, the presence of ≥3 foci was significant for an increased likelihood of developing DM in our subgroup analysis of PTC patients alone alongside a tumor size ≥2 cm, nodal metastasis and increased age.

The presence of DM had a substantial impact on survival in our cohort of WDTC patients (5-year and 10-year DSS of 71.0 and 46.9% respectively). Despite a multimodal approach to treatment, including RAI, EBRT and surgical resection of distant tumors, the overall survival remained significantly diminished in DM patients (OS 44.9% at 10 years, *p* < 0.001). These results emphasize the importance of early detection of patients that exhibit a greater risk of developing DM from WDTC.

The present study has several limitations. It is a retrospective chart review from a single institution. The sample size of the DM group is another limitation of our study. This is partly due to the rarity of DM in WDTC. Multi-center cohort studies are needed to mitigate sample size as a limitation considering the rarity of DM in thyroid carcinoma. Sample sizes required to generate more statistical power should roughly be greater than 68 DM patients according to our sample size estimates for a single-variable cox-proportional hazard model [22]. Given the small sample size, it is also difficult to separately analyze patients who developed DM throughout their treatment, compared to those who presented with DM. However, sparing positive resection margins and ETE, there were no significant differences between the groups. Future studies will focus on differentiating between the two groups. Future research is also investigating the influence of tumor driver mutations on the development of distant metastasis.

## Conclusion

WDTC patients with a tumor size ≥2 cm, NM and FTC on final pathology demonstrated a significantly increased propensity for developing DM. A subgroup analysis of PTC patients alone revealed increased age, presence of ≥3 foci, NM and tumor size ≥2 cm as significant contributors for the development of DM. Patients with DM have disproportionally higher frequencies of tall cell subtype of PTC, HCC and FTC. Our findings add to the existing literature on pathological risk factors for developing DM in WDTC, which will ultimately better inform the management of this patient group.

## Data Availability

Datasets are unavailable for this study as it would compromise patient privacy. However, further information regarding the data are available, within limits of patient privacy, upon request.

## Abbreviations

DM: Distant metastasis
NDM: Non-distant metastasis
WDTC: Well-differentiated thyroid cancer
PTC: Papillary thyroid cancer
FTC: Follicular thyroid cancer
HCC: Hurthle cell carcinoma
LVI: Lymphovascular invasion
NM: Nodal metastasis
TS: Tumor size
ETE: Extrathyroidal extension
PET: Positron emission tomography
CT: Computed tomography
OS: Overall survival
DSS: Disease-specific survival
HR: Hazard ratio
